# Nursing Problems in Ireland

**Published:** 1917-02-24

**Authors:** 


### THE EDITOR'S LETTER-BOX.

### Nursing Problems in Ireland.

*To the Editor of* THE HOSPITAL.

SIR,—In Ireland the College of Nursing will find its establishment threatened by storm and tempest, and in the early stages, at all events, response will be slow. Considering the stagnant condition of the nursing profession here this is not to be wondered at. Indeed, it would be astonishing if the situation were otherwise. The only Association of Nurses in Ireland is the Irish Nurses' Association, which has a nominal membership of three or four hundred out of some seven thousand. Its executive, which may be described as *semper idem*, consists of a handful of Dublin matrons, very capable and astute ladies no doubt, who have for years been discussing registration academically, who publish no reports of their proceedings, and who never consult the members as to their views or wishes. This Association, until the College started, was, in truth, moribund. It had no active propaganda outside of registration, and made no effort to organise the profession or raise its status. The result is that the general body of Irish nurses lack cohesion; they are without a public opinion, and are nervous and timid concerning any scheme which comes from without, and which is the subject of hostile criticism. The barrenness of achievement in the past by the I.N.A. is obvious to all. Hence, although the College will probably find recruiting slow, it possesses this advantage, that Irish nurses are unbiased, and if it is demonstrated in a practical manner that membership and progress are synonymous, the field will not prove unfruitful.

A WAR PRODUCT.

The fact that the College is a direct result of the war has not yet impressed itself on Ireland. Like the Mother Country and the Colonies, the nursing profession in the British Isles can never be the same as in ante-war days. Henceforth the Imperial ideal will be dominant, and we can no more tolerate ante-war statesmen than antediluvian statesmen. The College stands for the British Imperial ideal, and for Ireland to remain aloof would be to accentuate her insularity and to set a barrier to her progress. It is mainly to the younger generation that the ideal will appeal—to the nurse whose sphere and ambition are not bounded by the Irish Sea. When the war is over it is believed that emigration will receive a new stimulus. Thousands of young women will cross the Atlantic, and if the Royal British College of Nursing establishes a branch in Canada, the Irish nurse will not take long to recognise that membership possesses solid advantages. At the moment she cannot see beyond the region of jarring contention, of captious criticism, and personal prejudice.

THE MEDICAL ATTITUDE.

The physicians and surgeons of Ireland occupy a neutral position. In Dublin they side very largely with the executive of the I.N.A. simply because they know them and work with them. They are anxious as far as possible to help nurses to accomplish whatever they desire to accomplish. What they say is that if the nurses decide as to what they want they will get it, but the opinion prevails that they do not quite know their own mind, and this is very largely true. Many nurses of high intelligence who favour the College to-day are to-morrow desirous of a purely Irish College, and the following day they advocate the Registrationists' Bill. In the absence of any real organisation or of any public opinion in the Irish nursing world, this is a perfectly natural state of affairs, and only as a public opinion forms and develops will active recruiting to any school ensue. It is therefore up to the advocates of rival institutions, in being or in embryo, to make plain their programme, and to show by what means a successful issue may be reached.

PROPAGANDA.

The College has made a good beginning in securing as active supporters the matrons of three representative Dublin hospitals. The Mater Misericordiæ is a Catholic institution, the Adelaide Protestant, and the Royal City of Dublin unsectarian, so that it starts out with a non-party label written in large letters. This will substantially help in giving confidence to matrons and nurses all over the country, and, obviously, any other attitude would be fatal to progress. If a similar policy is pursued in Belfast, Cork, and elsewhere, and likewise with regard to medical men who may join the Irish Board, much will be done to silence destructive criticism. A strong forward programme is eminently desirable. In fact it is essential to success. Nurses will naturally expect a College to be an educational fountain. Those who suffer from a more or less ineffectual training will hail with delight a means of adding to their information in matters professional. They will, moreover, expect to be organised so as to enable them to obtain better remuneration and more liberal conditions of employment generally. If the College proves to be an active agent for the nurses' material benefit, opposition will quickly disappear, and Associations which have no other *raison d'être* than hostile criticism will find themselves without supporters. At the moment the air is thick with suspicion. The College is accredited with the base motive of endeavouring to filch from seven thousand impoverished Irish nurses seven thousand golden guineas; of contemplating the swamping of the trained nurse by letting loose a flood of V.A.D.s, while it is entirely forgotten that any registration arrangement must entail the payment of a fee, and that the V.A.D.s are at present quite as free to pose as trained nurses as any College can make them. All this suspicion will disappear if and when the College makes clear to the rank and file that its motto is "Progress," and that it possesses vital potentialities which other institutions lack. Much, consequently, will depend on the *personnel* of the Irish Board. It ought, if possible, to include representatives other than hospital matrons, whose position and reputation will guarantee to all concerned that the College stands in actual fact for all that it professes.—Yours faithfully,

IERNE.

